# Highly Efficient Removal of Organic Pollutants with HCO_3_^−^-Enhanced Ru(III)/NaClO Process

**DOI:** 10.3390/ijms26020677

**Published:** 2025-01-15

**Authors:** Yuhan Zhang, Guilong Peng, Yuting Yan, Xukun Meng, Wenwen Gong

**Affiliations:** 1 State Key Laboratory of Resource Insects, College of Sericulture, Textile and Biomass Sciences, Southwest University, Chongqing 400715, China; zyh74871231@163.com (Y.Z.); yyt18996371529@email.swu.edu.cn (Y.Y.); mengxukun2022@163.com (X.M.); 2 Westa College, Southwest University, Chongqing 400715, China; 3 Yibin Academy of Southwest University, Southwest University, Yibin 644001, China; 4 Institute of Quality Standard and Testing Technology, Beijing Academy of Agriculture and Forestry Science, Beijing 100097, China

**Keywords:** advanced oxidation processes, sodium bicarbonate, NaClO, Ru(III), Ru(V)=O

## Abstract

The design of efficient advanced oxidation processes (AOPs) in the presence of bicarbonate has long attracted considerable attention in the field of environmental catalysis. In this study, sodium bicarbonate (NaHCO_3_) as one of the most abundant substances in actual water, was introduced to a NaClO/Ru(III) system to enhance the removal of acid orange 7(AO7). NaHCO_3_ could significantly improve the removal efficiency of the Ru(III)/NaClO process in HCO_3_^−^ at a pH range of 6.9–10.0. Ru(V)=O was identified as a dominant reactive species involved in the degradation of pollutants in the NaHCO_3_/NaClO/Ru(III) system. HCO_3_^−^ interacts with Ru(III) to generate Ru(III)-HCO_3_^−^, which enhances the activation performance of Ru(III) under neutral or alkaline conditions. The removal of AO7 was significantly enhanced with increasing NaHCO_3_ concentration, and the rate constant increased more than 2-fold to 4-fold as NaHCO_3_ concentrations increased from 0 to 100 mM at pH 6.9 and 8.5. This study proposed a novel strategy to enhance the Ru(III)/NaClO process with environmentally friendly inorganic ligands and highlights its potential applications in the removal of pollutants.

## 1. Introduction

Advanced oxidation processes (AOPs) are based on the generation of highly reactive species, including radicals (e.g., hydroxyl radicals, superoxide anion radicals, sulfate radicals) [1,2] and non-radicals (e.g., singlet oxygen and high-valent metal–oxo species (HMOS)) [3,4] that show great potential in removing contaminants. Among those, HMOS exerts higher selectivity towards electron-rich substances, which in turn reduces the toxicity of such compounds [5,6]. In general, compared to radicals, HMOS have the advantages of a longer lifespan, higher steady-state concentration [7], and less susceptibility to being scavenged by non-target substrates such as natural organic matter and inorganic ions [8]. In addition, several studies have shown that HMOS, such as Fe(IV) and Co(IV), are typically formed in acid environments [6,9,10], while few studies have explored the formation of HMOS over a wide pH range, especially under neutral or alkaline conditions.

Up to now, various oxidants, such as ozone (O_3_) [11], hydrogen peroxide (H_2_O_2_) [12], persulfates [13,14], peracetic acid (PAA) [6], and periodate (IO_4_^−^) [15] have received considerable attention. Compared to the aforementioned oxidants, reports in the literature on the use of sodium hypochlorite (NaClO) oxidation technology for pollutant degradation are still limited. However, NaClO as an oxidant has unique advantages: it exhibits strong oxidizing properties over a wide pH range and is inexpensive and easy to produce [16]. Nevertheless, NaClO is not a strong oxidizing agent compared to Fenton oxidation [17], and generally requires a relatively long reaction time to achieve the high-efficiency removal of refractory compounds by ultraviolet (UV) activation [18]. In 2020, Liang et al. provided multiple evidence demonstrating that high-valent iron–oxo species (i.e., Fe(IV)) can be generated in the Fe(II)/HClO system under acidic conditions (at pH 2.0) via Equation (1) [19].

(1) $$Fe(II)+HClO\to {\left[Fe^{IV}O\right]}^{2+}+H^{+}+Cl^{-}$$

Ru(III) is a rare transition metal ion, which was employed to activate PAA for the removal of sulfamethoxazole (SMX) in a phosphate solution at pH 7.0, and the results suggested that the Ru(III)/PAA oxidation process was not affected by chloride and carbonate ions in the reaction solution [20]. Later, Zong et al. used Ru(III) as an activator to activate peroxymonosulfate (HSO_5_^−^, PMS) and found that the high-valent ruthenium–oxo species (i.e., Ru(V)=O) were the predominant reactive species in the Ru(III)/PMS system at pH 3.0 [21]. In this process, the inhibitory effects of inorganic ions and dissolved organic matters (DOMs) on SMX degradation were negligible. Bicarbonate ion (HCO_3_^−^) is one of the most abundant ions in actual water because of the high solubility of CO_2_ from the air. In the vast majority of AOPs, HCO_3_^−^ plays a significant inhibition role in the degradation of pollutants because it can quench free radicals to form weak reactive species [22,23,24]. However, there are a few reports on the enhanced removal of pollutants in the presence of HCO_3_^−^ [25]. Therefore, it is of great significance to investigate the effect of HCO_3_^−^ on catalytic activity in view of its practical applications.

Inspired by the above studies, we proposed a NaHCO_3_/NaClO/Ru(III) system with the expectation of achieving highly efficient contaminant removal under near-neutral pH and alkaline conditions, and until now little is known about the degradation of organic pollutants by this system. Previous studies have reported that HCO_3_^−^ can interact with metal ions, leading to significant changes in their reactivity [26,27]. We suspect that HCO_3_^−^ can significantly enhance the removal efficiency of contaminants in the activation of NaClO by Ru(III). Herein, the aim of the this study was to investigate the catalytic activity of NaHCO_3_/NaClO/Ru(III) for organic pollutants.

Acid orange 7 (AO7) is a representative substance among azo dyes, which is “carcinogenic, teratogenic and mutagenic” [28,29,30] and difficult to degrade, and was selected as the main test compound. Parameters influencing degradation using the present system were systematically examined, including NaClO and Ru(III) dosage, the concentration of AO7, and the concentration of HCO_3_^−^ under different pH values. Furthermore, the dominant reactive species in the NaHCO_3_/NaClO/Ru(III) system were identified, and the possible activation mechanisms were also elucidated.

## 2. Results and Discussion

### 2.1. The Degradation of AO7 in Different Metal Ion–NaClO Systems in the Presence of HCO_3_^−^

**Figure 1:**
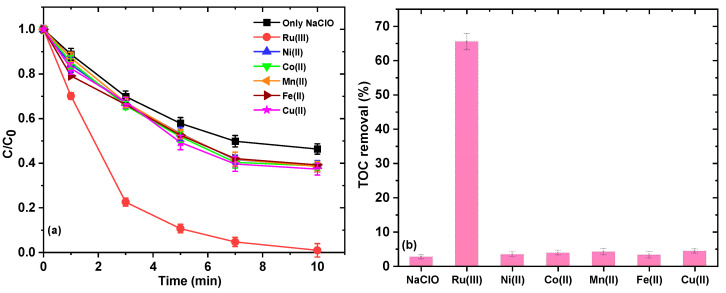
Removal of AO7 by NaClO activated by different metals (**a**) and TOC removal of AO7 (**b**). Experimental conditions: [AO7] = 0.03 mM; [NaClO] = 0.1 mM; [metal] = 10.0 μM; pH = 8.5 ± 0.1; [NaHCO_3_] = 10 mM; 10 mM borate buffer.

In the preliminary study, in addition to Ru(III), transition metals commonly employed in AOPs, such as Ni(II), Co(II), Mn(II), Fe(II), and Cu(II), were used for the activation of NaClO in the degradation of AO7, and then compared. Experiments were conducted by using a combination of 0.1 mM NaClO and 10 μM metal ions in the reaction solution that contained 10.0 mM NaHCO_3_ at pH 8.5 (Figure 1a). From the experimental results, it can be seen that only NaClO can remove 54% of AO7, which was caused by the direct oxidation of AO7 due to the strong oxidizing property of NaClO. The poor activation of NaClO by Ni(II), Co(II), Mn(II), Fe(II), and Cu(II) may be attributed to the formation of metal hydroxides in the presence of HCO_3_^−^ at alkaline pH 8.5. Overall, Ru(III) exhibited superior properties to other transition metals in the activation of NaClO for AO7 removal under the studied experimental conditions (i.e., 10.0 mM NaHCO_3_ at pH 8.5). The corresponding spectra for the removal of AO7 with and without Ru(III) activation of NaClO are shown in Figure S2. As shown in Figure 1b, although AO7 can be removed by 40% in the system with only NaClO, the TOC removal efficiency of AO7 is only 2.6% within 10 min of reaction. However, in the Ru(III)/NaClO system, the TOC removal efficiency of AO7 can reach 65.5%. That is to say, NaClO alone would just lead to the decomposition of the AO7 molecules, and it cannot provide sufficient oxidation capacity for the mineralization of residual organic molecules.

### 2.2. Effect of Reaction Parameters

**Figure 2:**
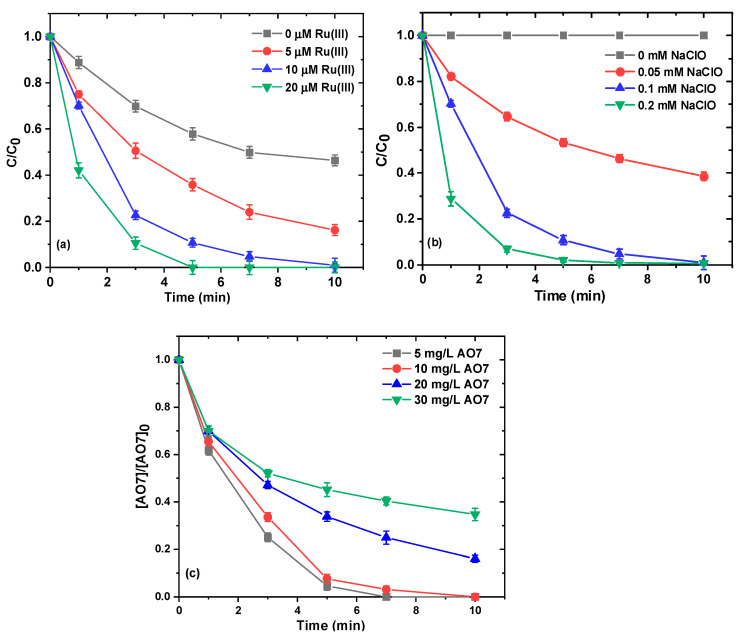
Effect of concentration of Ru(III) (**a**), NaClO (**b**), and AO7 (**c**) on removal of AO7. Experimental conditions: [NaClO]_0_ = 0.1 mM for (**a**,**c**); [Ru(III)]_0_ = 0.01 mM for (**b**,**c**); [AO7]_0_ = 0.03 mM for (**a**–**c**); [NaHCO_3_] = 10 mM for (**a**–**c**); 10 mM borate buffer for (**a**–**c**); pH = 8.5 ± 0.1.

Figure 2a presents the influence of different dosages of Ru(III) on the degradation efficiency of AO7. When the dosage of Ru(III) increased from 0 to 20 μM, the degradation efficiency of AO7 increased from 54% to 100% within 10 min, and the corresponding pseudo-first-order degradation rate constants (*k_obs_*) increased from 0.0799 min^−1^ to 0.7427 min^−1^. This suggests that Ru(III) can activate NaClO, resulting in faster oxidation of AO7 in water, and higher doses of Ru(III) can generate more reactive species to promote the removal of AO7.

To investigate the effect of NaClO concentrations on AO7 removal, experiments were performed at a constant Ru(III) concentration of 10.0 μM and different NaClO concentrations (i.e., 0, 0.05, 0.1, and 0.2 mM) (Figure 2b). The removal efficiency of AO7 significantly increased with the increasing NaClO concentration in the presence of Ru(III). The removal of AO7 increased from 61% at 0.05 mM NaClO to 100% at 0.2 mM. Notably, in the absence of NaClO (NaClO = 0.0 mM), Ru(III) alone had no role in the removal of AO7.

The influence of the initial concentration of AO7 was investigated by varying its initial concentration from 5 mg/L to 30 mg/L. As shown in Figure 2c, the removal efficiency of AO7 decreased as the initial AO7 concentration increased. When the initial concentration of AO7 increased from 5 to 30 mg/L, the removal extent of AO7 could reach 95% and 98% with lower AO7 concentrations of 5 and 30 mg/L, respectively. AO7 removal efficiency changed from 100% to 65% within 10 min over the whole initial concentration range (5–30 mg/L), and the corresponding *k_obs_* values decreased from 0.603 min^−1^ to 0.0966 min^−1^, demonstrating the superior AO7 removal performance at various concentration levels in the NaHCO_3_/NaClO/Ru(III) process. This phenomenon could be attributed to the fact that the amount of reactive species produced remained constant at a given dose of NaClO and Ru(III). In addition, at a lower initial AO7 concentration, a higher ratio of reactive species to AO7 provided a greater likelihood that AO7 molecules would be attacked, resulting in a higher removal rate.

### 2.3. Contributions of Different Reactive Species

**Figure 3:**
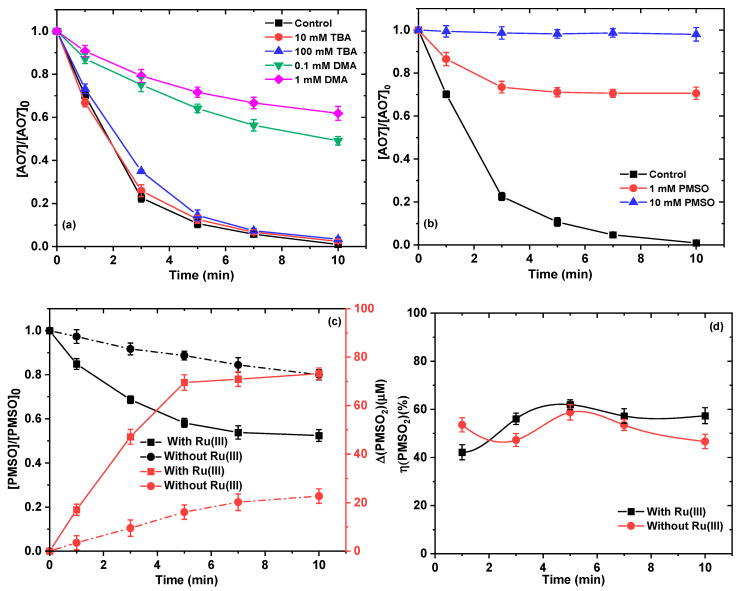
Effect of TBA and DMA (**a**) and PMSO (**b**) on AO7 removal; oxidation of PMSO and production of PMSO_2_ (**c**); and calculated η(PMSO_2_) values (**d**). Experimental conditions: [NaClO]_0_ = 0.1 mM; [Ru(III)]_0_ = 0.01 mM; [AO7]_0_ = 0.03 mM; [PMSO]_0_ = 0.03 mM for (**c**); [NaHCO_3_] = 10 mM for (**a**–**c**); 10 mM borate buffer for (**a**–**c**); pH = 8.5 ± 0.1.

Based on the previous reports, NaClO could activate iron to produce hydroxyl radicals (^•^OH) and chlorine radicals (ClO•), which were responsible for degrading various pollutants [31]. Considering the similarity, quenching experiments were conducted by adding various scavengers to explore the involved reactive species in the NaHCO_3_/NaClO/Ru(III) system. Tert-butyl alcohol (TBA) is an effective scavenger for ^•^OH, ClO^•^ and Cl^•^ ($k_{{•}_{OH,TBA}}=6.0\times 10^{8}\;M^{-1}s^{-1}$, $k_{ClO^{•},TBA}=1.3\times 10^{7}\;M^{-1}s^{-1}$, and $k_{Cl^{•},TBA}=3.0\times 10^{8}\;M^{-1}s^{-1}$) (Table S2), but it has a negligible scavenging effect on carbonate radical anion ($k_{{CO_{3}}^{•-},TBA}=9.6\times 10^{4}\;M^{-1}s^{-1}$) (Table S2). As can be seen from Figure 3a, the addition of TBA (10–100 mM) had almost no effect on the removal of AO7, indicating that ^•^OH, ClO^•^, and/or Cl^•^ may not be the reactive species in the NaHCO_3_/NaClO/Ru(III) system. In view of the presence of CO_3_^2−^/HCO_3_^−^ and their enhancing effect in the reaction solution, we deduced that CO_3_^•−^ might play an indispensable role in the removal process of AO7. To validate our hypothesis, N, N-dimethylaniline (DMA) was used as the effective scavenger for CO_3_^•−^ ($k_{{CO_{3}}^{•-},DMA}=1.8\times 10^{9}\;M^{-1}s^{-1}$) [32] to investigate its contribution level. As can be seen in Figure 3a, when 0.1 and 1.0 mM DMA was added to the reaction solution, the removal of AO7 was reduced to 51% and 38%, respectively, suggesting that CO_3_^•−^ may be involved in the NaHCO_3_/NaClO/Ru(III) system. This shows that Fe(II) can react with ClO^−^ to form [Fe^IV^O]^2+^ and the similarity between Fe(IV) and Ru(V)=O, Ru(V)=O may be involved in the present system. Here, phenyl methyl sulfoxide (PMSO) was used as a scavenger to reveal the role of high-valent Ru–oxo (e.g., Ru(V)=O) species [33] in the NaHCO_3_/NaClO/Ru(III) system. As depicted in Figure 3b, when PMSO (10 mM) was introduced into the reaction solution, the removal of AO7 is almost completely quenched. This demonstrated that Ru(V)=O played an important role of Ru(V)=O in the removal of AO7. To further verify the role of Ru(V)=O in the NaClO/Ru(III)-CO_3_^2−^/HCO_3_^−^ system, we monitored the conversion of PMSO to methyl phenyl sulfone (PMSO_2_). If the reaction involves a high-valent metallic oxygen species, PMSO can be oxidized to the corresponding PMSO_2_, which is significantly different from the free radical-mediated pathway. As can be seen from the chromatograms in Figure S3, the peak intensities of PMSO_2_ formed in the presence of NaHCO_3_ were much more significant than that in the absence of NaHCO_3_. Similar results were obtained in the study of Huang et al., who proposed that HCO_3_^−^ is an important complexing ligand for some metals such as cobalt and manganese, and that the complexation between them can lead to the production of high-valent metal–oxo intermediates [25].

According to our recent study, NaClO can directly oxidize phenyl methyl sulfoxide (PMSO) and transform to methyl phenyl sulfone (PMSO_2_) [14]. As can be seen in Figure 3c, 20% of PMSO can be directly oxidized by NaClO and generated only 22.7 μM PMSO_2_ within 10 min. However, in the presence of Ru(III), the degradation of PMSO can reach 48% within the same reaction time, and 73.1 μM PMSO_2_ could be generated. The yield of PMSO_2_ (i.e., η(PMSO_2_), which is defined as the conversion ratio of PMSO to PMSO_2_, was quantified to be 47–59% and 42–62% for the NaClO/NaHCO_3_ and NaHCO_3_/NaClO/Ru(III) systems (Figure 3d), respectively. These results are different from our previous study [14], where the yield of PMSO_2_ obtained through the direct oxidation of PMSO with NaClO was close to 100%. This may be caused by the presence of HCO_3_^−^/CO_3_^2–^ in the reaction system, and carbonate radicals may be involved in the conversion of PMSO; more in-depth mechanisms still require further study in future work.

### 2.4. Effect of Concentration of HCO_3_^−^ Under Different pH Values

**Figure 4:**
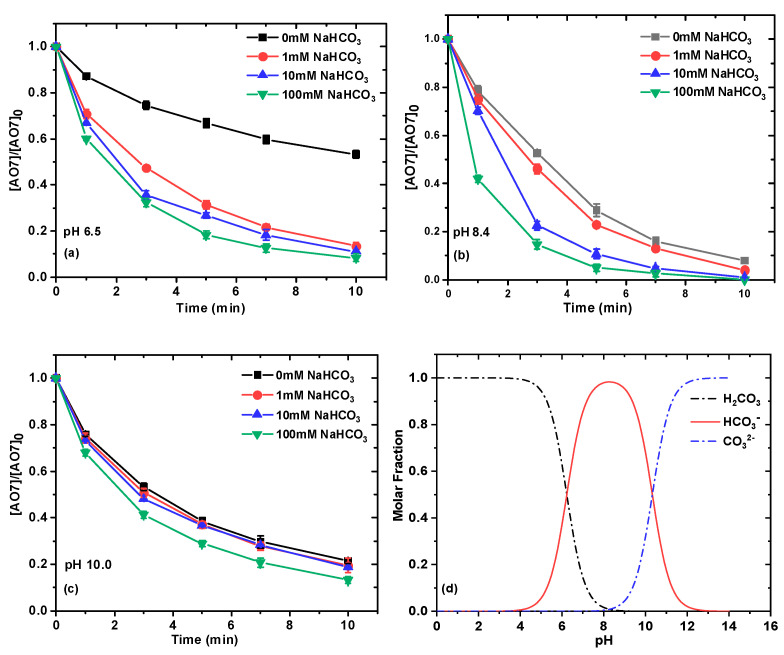
Effect of NaHCO_3_ concentration on removal of AO7 at pH 6.9 (**a**), 8.5 (**b**), and 10.0 (**c**); distribution of different species of H_2_CO_3_ (**d**). Experimental conditions for (**a**–**c**): [NaClO]_0_ = 0.1 mM; [Ru(III)]_0_ = 0.01 mM; [AO7]_0_ = 0.03 mM; [NaHCO_3_] = 10 mM; 10 mM borate buffer; pH = 8.5 ± 0.1.

As is well known, carbonic acid is a diprotic acid with two pK_a_ values (pK_a1_ = 6.4; pK_a2_ = 10.3) [34], so carbonate has three species in water, namely H_2_CO_3_, HCO_3_^−^, and CO_3_^2–^, and their distribution fractions are highly pH-dependent. In order to gain a deeper comprehension of their speciation on the effect of AO7 removal, the experiments were carried out under different pH values in the presence of different concentrations of HCO_3_^−^/CO_3_^2–^. The reaction solution was first adjusted to different pH values with 0.1 M NaOH or H_2_SO_4_ in a 10 mM borate buffer, then Ru(III) and NaClO were added, respectively, and the initial pH was recorded after the addition of NaClO. The kinetics for AO7 removal in NaHCO_3_/NaClO/Ru(III) under different pH values can be seen in Figure 4a–c. Obviously, the addition of NaHCO_3_ would increase the removal of AO7 when the pH values increased from 6.9 to 10.0. As depicted in Table 1, the removal efficiencies of AO7 within 10 min increased from 46.74% to 91.65%, from92.65% to 100%, and from 78.48% to 86.83% for pH values of 6.9, 8.5, and 10.0 with the addition of 0 mM to 100 mM NaHCO_3_, respectively. Additionally, data fitting showed that AO7 removal could be described by pseudo-first-order kinetics, where the rate constant values (*k_obs_*) increased from 0.06122 to 0.0.2454 min^−1^, from 0.2577 to 0.5169 min^−1^, and from 0.1517 to 0.1968 min^−1^ as the NaHCO_3_ concentration increased from 0 mM to 100 mM for pH values of 6.9, 8.5, and 10.0, respectively. The removal rate of AO7 is always much faster at pH 8.5 than that of pH 6.9 and pH 10, which may be due to the fact that HCO_3_^−^ is the dominant carbonate species at pH 8.5. According to the distribution fractions of H_2_CO_3_ (Figure 4d), the molar percentages of HCO_3_^−^ at pH 6.9, 8.5, and 9.0 are 76%, 98%, and 67%, respectively. We can also observe from Table 1 that, under the same reaction time and NaHCO_3_ concentration, the removal efficiency of AO7 follows the order of pH 8.5 > pH 6.9 > pH 10.0, which is consistent with the molar percentage order of HCO_3_^−^. These results suggested that the addition of NaHCO_3_ can significantly enhance the removal efficiency and rate of AO7 in the NaHCO_3_/NaClO/Ru(III) system, which may be due to the introduction of HCO_3_^−^ rather than CO_3_^2−^. It is worth noting that the increase of NaHCO_3_ concentration had little effect on the removal rate of AO7 at pH 10, indicating that the increase of HCO_3_^−^/CO_3_^2–^ had little effect on the reactive species under this condition. Additionally, HClO has a pK_a_ of 7.5 [35], and from the molar distribution of HClO, it can be concluded that the molar percentages of ClO^−^ are 20%, 91%, and 100% at pH 6.9, 8.5, and 10.0, respectively. Therefore, these results suggest that the concentration of ClO^−^ species may have less of a role than those of HCO_3_^−^ species in the removal of AO7.

In Figure 3c above, we obtained the result that the addition of Ru(III) could greatly increase the generation of PMSO_2_ in the NaHCO_3_/NaClO/Ru(III) system. Based on these results, the degradation of PMSO experiments were also conducted under different pH values (i.e., 6.9, 8.5, and 10.0) in the presence and in the absence of HCO_3_^−^/CO_3_^2–^. As shown in Figure S4a,b, PMSO_2_ was significantly generated at pH 6.9 and pH 8.5, and the amount of PMSO_2_ generated in the presence of NaHCO_3_ was always higher than that in the absence of NaHCO_3_, indicating that more Ru(V)=O was generated in the presence of NaHCO_3_. However, no significant PMSO_2_ formation was observed (Figure S4c), which may be due to the hydrolysis and precipitation of Ru(III) at higher pH values, thereby reducing the concentration of dissolved Ru(III) available for NaClO activation. The above results implied that the introduction of HCO_3_^−^ induced the production of more Ru(V)=O in the NaHCO_3_/NaClO/Ru(III) system, which was consistent with the results obtained in the previous Co-based PMS/HCO_3_^−^ system [25].

Since the *k_obs_* value is highly affected by the metal ion and NaClO dose, an evaluation parameter, *K*, is defined here (Equation (2)) with reference to the previous method [33] to more accurately assess the performance of the NaHCO_3_/NaClO/Ru(III) system. The *K* values of the comparison systems were calculated based on the data provided by the corresponding reports, i.e., *k_obs_* values and the dosages of metals and oxidants. As displayed in Table 1, the *K* values in the NaHCO_3_/NaClO/Ru(III) system were 61.2 to 245.4 min^−1^ mM^−2^, 257.7 to 516.8 min^−1^ mM^−2^, and 151.7 to 196.8 min^−1^ mM^−2^ at pH 6.9, 8.5, and 7.0, respectively, which were two orders of magnitude higher than those in all comparison systems (Table S3), further suggesting that the present studied system is a high-performance AOP.

(2) $$K=\frac{k_{obs}}{{\left[Ru(III)\right]}_{0}{\left[NaClO\right]}_{0}}$$

### 2.5. Impact of Common Anions

**Figure 5:**
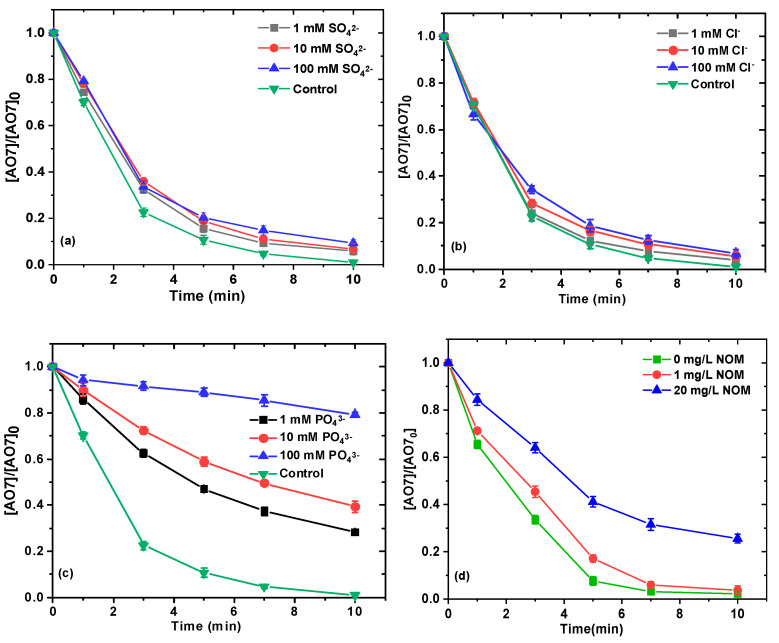
Effect of SO_4_^2−^ (**a**), Cl^−^ (**b**), PO_4_^3−^ (**c**) as well as NOM (**d**) on removal of AO7. Experimental conditions: [NaClO]_0_ = 0.1 mM; [Ru(III)]_0_ = 0.01 mM; [AO7]_0_ = 0.03 mM; [NaHCO_3_] = 10 mM; 10 mM borate buffer; pH = 8.5 ± 0.1.

In the present study, SO_4_^2−^, Cl^−^, and PO_4_^3−^ were selected to investigate their effects on the removal of AO7 in the NaHCO_3_/NaClO/Ru(III) system, as they are ubiquitous in various natural waters and wastewaters, and their presence may promote, inhibit, or have no obvious effect on the removal of contaminants from water, depending on the situation [22,36,37,38]. As depicted in Figure 5a,b, the removal of AO7 was not significantly affected in the presence of SO_4_^2−^ and Cl^−^ at concentrations ranging from 1 to 100 mM. These results showed that SO_4_^2−^ and Cl^−^ had little effect on the removal of AO7, implying that the dominant reactive species Ru(V)=O was not insensitive to SO_4_^2–^ and Cl^−^. These phenomena can also be attributed to their low oxidation/reduction reactivity with the primary oxidant Ru(V)=O [21]. In contrast, the removal of AO7 was significantly inhibited in the presence of the same concentration of PO_4_^3–^ (Figure 5c). It was also reported that PO_4_^3–^ can act as a chelating agent to form complexes with Ru(III), and cause a strong inhibitory effect in the activation of periodate by Ru(III) [33].

Moreover, organic compounds are widely present in waters and wastewaters in the form of natural organic matter (NOM) and may interact with the target contaminant and/or reactive species in AOPs, due to the presence of carboxyl groups and phenolic hydroxyls [39,40]. In this study, humic acid sodium was used as a representative of large molecular weight NOM. As shown in Figure 5d, the removal of AO7 was inhibited with the addition of NOM from 1 to 20 mg/L. When the NOM concentration increased from 0 to 20 mg/L, the removal efficiency of AO7 decreased from 100% to 74%, indicating that NOM plays a detrimental role in the reaction process.

### 2.6. Degradation of Other Pollutants in *NaHCO_3_/NaClO/Ru(III)* System

**Figure 6:**
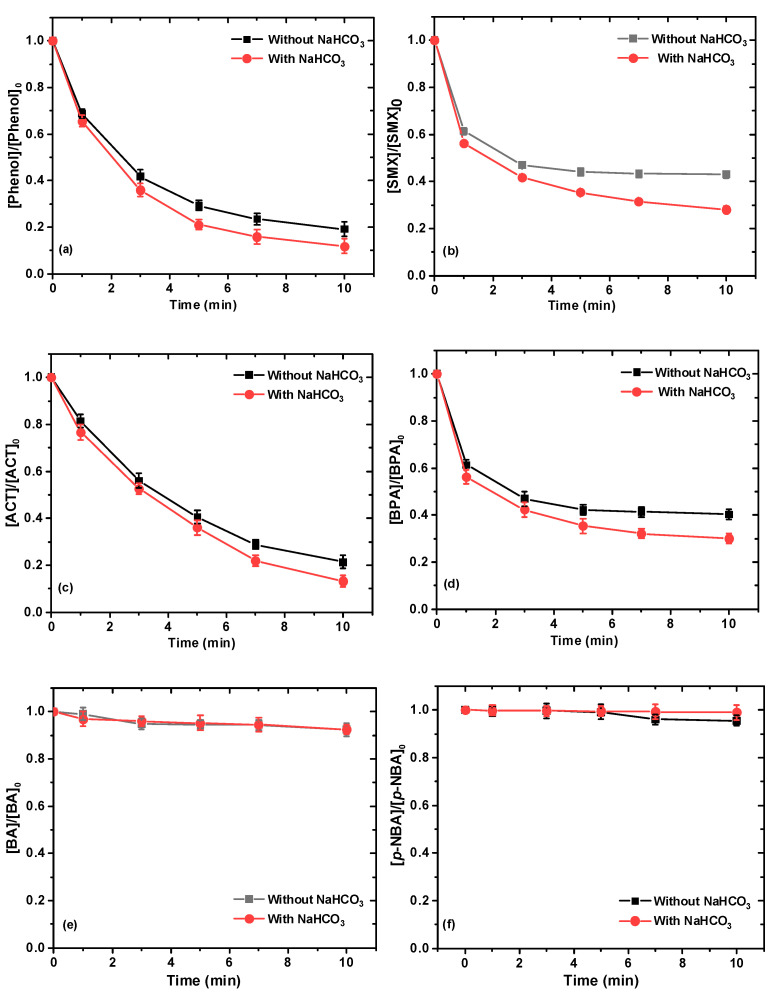
Degradation of phenol (**a**), SMX (**b**), ACT (**c**), BPA (**d**), BA (**e**), *p*-NBA (**d**), BA (**e**), and *p*-NBA (**f**) in presence and in absence of NaHCO_3_. Experimental conditions: [NaClO]_0_ = 0.1 mM; [Ru(III)]_0_ = 0.01 mM; [phenol]_0_ = [SMX]_0_ = [ACT]_0_ = [BPA]_0_ = [BA]_0_ = [*p*-NBA]_0_ = 0.03 mM; [NaHCO_3_] = 10 mM; 10 mM borate buffer; pH = 8.5 ± 0.1.

In addition to AO7, six other representative organic pollutants, including acetaminophen (ACT), bisphenol A (BPA), sulfamethoxazole (SMX), phenol, benzoic acid (BA), and *p*-nitrobenzoic acid (*p*-NBA), were also used as target pollutants to examine the catalyst performance of Ru(III) in the presence and absence of HCO_3_^−^/CO_3_^2–^ under the condition of [NaClO]_0_ = 0.1 mM; [pollutants]_0_ = 10 mg/L; [Ru(III)]_0_ = 10 μM; and pH = 8.5. The chemical properties and structures of these studied compounds are provided in Table S4. As seen in Figure 6a–f, satisfactory removal efficiencies for phenol, SMX, ACT, and BPA could be achieved within 10 min, and their removal performance in the presence of HCO_3_^−^/CO_3_^2–^ was always better than in the absence of HCO_3_^−^/CO_3_^2–^. The enhanced removal effect was due to the generation of more Ru(V)=O reactive species in the presence of HCO_3_^−^/CO_3_^2–^, which was confirmed by our aforementioned research results. However, there was no significant efficacy for the removal of BA and *p*-NBA in the presence of HCO_3_^−^/CO_3_^2–^ and in the absence of HCO_3_^−^/CO_3_^2–^, implying the selective reactive species Ru(V)=O generated in the NaHCO_3_/NaClO/Ru(III) process. High-valent iron–oxo species (i.e., Fe(IV)) are selective oxidants that are unreactive to organic pollutants containing electron-withdrawing groups such as nitro (-NO_2_) or carboxyl (-COOH), and exhibit an opposite tendency towards electron-rich groups such as amide (-NH_2_) and hydroxyl (-OH) [15]. Therefore, the poor removal of BA and *p*-NBA can be attributed to the fact that the Ru(V)=O species is a selective oxidant and it is unreactive to organic pollutants containing electron-withdrawing groups, which is consistent with the results of previous Ru(III)-activated PMS [21].

### 2.7. Comparison with Other Advanced Oxidation Systems

**Figure 7:**
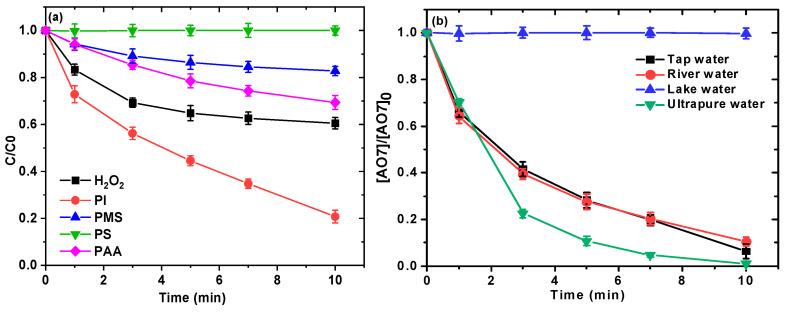
Removal of AO7 with various oxidants activation of Ru(III) (**a**) and removal of AO7 in different water (**b**). Experimental conditions: [NaClO]_0_ = 0.1 mM; [Ru(III)]_0_ = 0.01 mM; [AO7]_0_ = 0.03 mM for (**a**,**b**); [NaHCO_3_] = 10 mM for (**a**); 10 mM borate buffer for (**a**); pH = 8.5 ± 0.1.

To test the activation performance of Ru(III) against other oxidants, we also evaluated the efficacy of activating Ru(III) with various oxidants such as H_2_O_2_, sodium periodate (PI), peroxymonosulfate (PMS), peroxydisulfate (PDS), and peracetic acid (PAA) for the removal of AO7 at pH 8.5 in the presence of 10 mM HCO_3_^−^/CO_3_^2–^. As shown in Figure 7a, the NaHCO_3_/PI/Ru(III) process provided a very high removal efficiency of ~80% within 10 min with *k_obs_* of 0.1465 min^−1^. Li et al. [33] have confirmed the high activation performance of Ru(III) towards PI by degrading carbamazepine in the Ru(III)/PI system; the results showed that Ru(III) could activate PI and produce high-valent Ru–oxo species in the initial pH range of 3.0–11.0. Almost no significant AO7 removal could be observed for PS, suggesting that Ru(III) may not be able to effectively trigger PS to produce effective reactive species for AO7 removal under current conditions. In addition, about 39%, 17%, and 31% were removed within 10 min for H_2_O_2_, PMS, and PAA, respectively. H_2_O_2_ activated by Ru(III) has not been reported; however, recent studies have shown that PMS and PAA could be activated by Ru(III) under certain conditions. For example, PMS can be activated by 0.15 mM Ru(III) at pH 3.0 for the degradation of various organic pollutants [21]. Ru(III) was employed to activate PAA for the degradation of micro-pollutants, and 95.5% of 20 μM SMX could be removed in the phosphate solution at pH 7.0 with 0.2 mM Ru(III) activating 0.1 mM PAA within 4 min.

### 2.8. Evaluation in Actual Water

To investigate the practical feasibility of the NaHCO_3_/NaClO/Ru(III) process in actual water, the efficacy of this process in different water matrices, including river water, tap water, and lake water, was also evaluated in this study. As shown in Figure 7b, 90% and 94% of AO7 removal were achieved in river water and tap water, respectively, which were slightly lower compared to that in ultrapure water (100%) within 10 min. However, the removal of AO7 in the lake water could be ignored. The poor removal of AO7 degradation in the lake water sample compared to river water and tap water might be attributed to the presence of the higher concentration of dissolved natural organic matter and inorganic ions (PO_4_^3−^). Based on the concentration of each component in the three actual water matrices (Table S5), it was postulated that NOM and PO_4_^3−^ are the primary factors inhibiting the removal of AO7 in the lake water.

As described in Section 2.5, the effect of SO_4_^2−^ (1–100 mM) and Cl^−^ (1–100 mM) on AO7 removal was not significant. Therefore, the effect of these anions on AO7 removal kinetics is negligible, as the concentrations in all actual water were lower than those aforesaid concentrations. Since both NOM and PO_4_^3−^ can significantly inhibit the removal kinetics of AO7 (Figure 5c,d), it is reasonable to deduce that when the process is employed to remove organic contaminants from real water, the water is effectively pretreated to remove NOM and PO_4_^3−^, which is optimal for achieving significant removal efficiency.

### 2.9. Proposed HCO_3_^−^-Enhanced Mechanism

Based on the above results and discussion, a possible HCO_3_^−^-enhanced reaction mechanism in the NaHCO_3_/NaClO/Ru(III) process was proposed. Firstly, NaClO was rapidly activated by Ru(III) to generate Ru(V)=O via Equation (3); the target pollutant was then degraded by the generated Ru(V)=O. Subsequently, HCO_3_^−^ can interact with Ru(III) to form a Ru(III)-HCO_3_^−^ complex (Equation (4)). Then, the formed Ru(III)-HCO_3_^−^ complex could be rapidly oxidized by ClO^−^ to produce Ru(V)=O (Equation (5)), which can further work together with the formed Ru(V)=O in Equation (3) to enhance the removal of pollutants. The introduction of HCO_3_^−^ enhanced the removal of pollutants by the generation of more Ru(V)=O, which was demonstrated by the fact that more PMSO_2_ could be produced in the presence of HCO_3_^−^ than in the absence of HCO_3_^−^. The results in Section 2.4 above show that increasing the concentration of NaHCO_3_ from 0 to 100 mM can increase the removal of AO7, particularly pronounced at pH 6.9 and 8.5, which is attributed to HCO_3_^−^ becoming the predominant species in this pH range.

**Figure 8:**
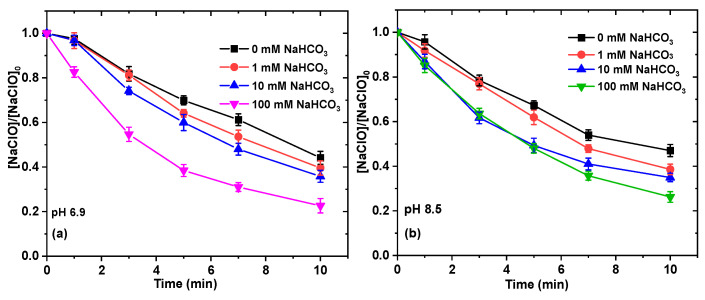

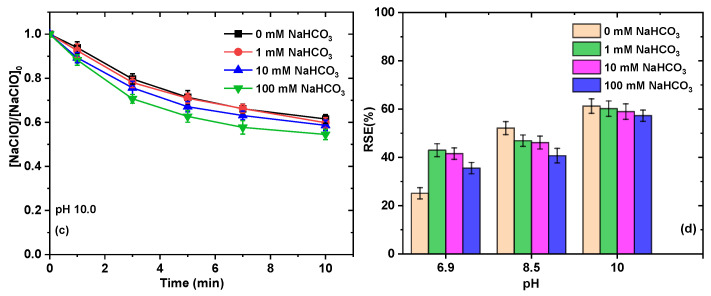
Effect of NaHCO_3_ concentration on consumption of NaClO in NaHCO_3_/NaClO/Ru(III) process at pH 6.9 (**a**), 8.5 (**b**), and 10.0 (**c**); utilization efficiency of NaClO to remove AO7 with NaHCO_3_/NaClO/Ru(III) process at different NaHCO_3_ concentrations at pH 6.9, 8.5, and 10.0 (**d**).

In a recent study, the introduction of HCO_3_^−^ could enhance the degradation of emerging contaminants (ECs) in the Mn(II)/PAA process. This enhanced degradation of contaminants can be attributed to the fact that HCO_3_^−^ can act as a ligand in conjunction with Mn(III) and/or Mn(V) to produce complexes, thereby prolonging the exposure time of Mn(III) and/or Mn(V) in the NaHCO_3_/Mn(II)/PAA process [27]. To better evaluate the utilization efficiency of NaClO, the consumption of NaClO in the process of NaHCO_3_/NaClO/Ru(III) was monitored at different concentrations of NaHCO_3_ at different pH values to calculate the reaction stoichiometric efficiency (RSE; Equation (6) shows the ratio of the removed AO7 to the consumed NaClO) [41] (Figure 8a–c). As shown in Figure 8d, the addition of NaHCO_3_ can decrease the utilization efficiency of NaClO, and the RSE values decreased from 43% to 36%, from 53% to 45%, and from 61% to 57% as the concentration of NaHCO_3_ increased from 1 to 100 mM at pH 6.9, 8.5, and 10.0, respectively. The RSE values increase with increasing pH, which may be related to the molar percentage of ClO^−^. According to the distribution of different species of HClO (Figure S5), the molar fractions of ClO^−^ at pH 6.9, 8.5, and 10.0 are 20%, 91%, and 100%, respectively, indicating that more ClO^−^ is more favorable for the progress of Equations (3) and (5). These results also suggest that NaHCO_3_ is more suitable as a ligand-stabilized intermediate Ru(V)=O under alkaline conditions, reducing the ineffective consumption of the intermediates Ru(V)=O and NaClO.

(3) $$Ru(III)+ClO^{-}\to Ru(V)=O+Cl^{-}$$

(4) $$Ru(III)+{HCO}_{3}^{-}\to Ru(III)-HCO_{3}^{-}$$

(5) $$Ru(III)-HCO_{3}^{-}+ClO^{-}\to Ru(V)=O+Cl^{-}+CO_{3}^{2-}+H^{+}$$

(6) $$RSE(\%)=\frac{\Delta AO7}{\Delta NaClO}\frac{\left(mol\right)}{\left(mol\right)}\times 100\%$$

## 3. Materials and Methods

### 3.1. Materials

All chemical reagents and organic solvents used in this study were at least analytical grade and used directly without further purification. Acetonitrile (>99.5%), methanol (MeOH, 99.9%), ethanol (EtOH, 99.9%), phenyl methyl sulfoxide (PMSO, 98%), methyl phenyl sulfone (PMSO_2_, 98%), potassium peroxymonosulfate (PMS, KHSO5·0.5KHSO4·0.5K2SO4, ≥47% KHSO_5_ basis), sodium persulfate (Na_2_S_2_O_8_, 99%), sodium periodate (NaIO_4_, ≥99.8%), hydrogen peroxide solution (H_2_O_2_, 30% wt. in H_2_O), sodium hydroxide (NaOH, ≥98%), sulfuric acid (H_2_SO_4_, 96%), sodium hypochlorite(NaClO, ≥98%), sodium bicarbonate (NaHCO_3_, ≥99.8%) ruthenium(III) chloride hydrate (RuCl_3_•xH_2_O, 35–42% Ru basis), nickel sulfate hexahydrate (NiSO_4_•6H_2_O, ≥98%), cobalt(II) chloride hexahydrate (CoCl_2_•6H_2_O, ≥97%), manganese(II) sulfate monohydrate (MnSO_4_•H_2_O), iron(II) sulfate heptahydrate (FeSO_4_•7H_2_O, ≥99.9%), and cupric sulfate (CuSO_4_, ≥99%) were purchased from Aladdin (Shanghai, China). Peracetic acid (PAA, 14.4%) was purchased from Tianjin Oubokai Chemical Co., Ltd. (Tianjin, China).

AO7 (>98%), acetaminophen (ACT, 99%), bisphenol A (BPA, ≥99.8%), benzoic acid (BA, >99.5%), *p*-nitrobenzoic acid (*p*-NBA, ≥99%), phenol (≥99%), sulfamethoxazole (SMX, ≥98%), tert-butanol (TBA, >99.5%), humic acid sodium (NOM, AR), sodium chloride (NaCl, >99.5%), sodium phosphate tribasic dodecahydrate (Na_3_PO_4_•12H_2_O, ≥98%), and other chemicals were purchased from Sigma-Aldrich (Shanghai, China). All the stock and working solutions were prepared in ultrapure water produced by a Milli-Q system (EMD Millipore, Billerica, MA, USA).

### 3.2. Experimental Procedure

The initial concentration (C_0_) of the organic pollutants was fixed at 0.03 mM, except in experiments investigating the effect of the initial concentration of AO7. All degradation experiments were performed in a 150 mL glass conical flask at a controlled temperature of 25 °C under continuous magnetic stirring, using a constant temperature water bath magnetic stirrer (HH-6A, Suzhou Weier experimental supplies Co., Ltd., Suzhou, China). The target pollutant solution, the NaClO solution, and the NaHCO_3_ solution were sequentially added to a 150 mL glass conical flask to achieve the predetermined concentrations. Then, the reaction was triggered by introducing 10 μM Ru(III) into the solution, except in the studies investigating the effect of the dosages of Ru(III). At the specified reaction time intervals, 2 mL of the sample was withdrawn and the concentration of AO7 was determined immediately. For other analyses, the samples were quenched with 0.2 mL Na_2_S_2_O_3_ (10 mM). During the experiment, borate buffer was added to stabilize the pH of the Ru(III)/NaClO process. The pH of the reaction solution was carefully adjusted using NaOH or H_2_SO_4_ solutions (0.1 M). The effect of borate buffer on the Ru(II)/NaClO process and the NaHCO_3_/Ru(II)/NaClO process was negligible (Figure S1). All experiments were repeated at least twice.

### 3.3. Analytical Methods

The concentration of AO7 was measured at their characteristic λ_max_ of 484 nm using a DR6000 UV-vis spectrophotometer (HACH, Loveland, CO, USA). The concentrations of other organic compounds were measured by high-performance liquid chromatography (HPLC, UltiMate 3000, Thermo Scientific, Germering, Germany) equipped with a diode array detector. The organic compounds were separated with a C18 column (Agilent, 5 μm, 250 × 4.6 mm). The detailed analytical parameters for HPLC analysis are shown in Table S1**.** Solution pH was measured with an FE28-Standard pH meter (Mettler Toledo, Shanghai, China).

## 4. Conclusions

In the present study, the removal of AO7 by the Ru(III)/NaClO system provided a novel oxidation system to efficiently degrade pollutants in water containing HCO_3_^−^/CO_3_^2–^ buffers. The results demonstrated that 100% of AO7 could be removed in the NaHCO_3_/NaClO/Ru(III) system within 10 min with a high rate constant of 0.4585 min^−1^. Significantly, when HCO_3_^−^ was introduced into this system, it was found that the removal of AO7 was remarkably enhanced, and the rate constant increased by more than 2- to 4-fold at pH 6.9 and 8.5. Interestingly, the key factor responsible for improved catalytic oxidation was the novel reactive species, Ru(V)=O, which was confirmed by using PMSO as a probe compound. This oxidation system was further investigated by removing 50 μM of various pollutants at pH 8.5 using 0.1 mM NaClO and 10 μM Ru(III). Satisfactory removal efficiencies for electron-rich groups compounds such as phenol, SMX, ACT, and BPA were obtained; however, no obvious removal of electron-rich organic electron-withdrawing group compounds such as BA and *p*-CBA was determined, indicating the dominant role of high-valent Ru–oxo (e.g., Ru(V)=O) species in the reaction system. Using novel oxidation technology to create a complex of the NaClO/Ru(III) process and HCO_3_^−^ showed a wide application prospect in pollutant degradation, and has the potential for further designing effective oxidation processes of the treatment of actual wastewater under neutral or alkaline conditions.

## Figures and Tables

**Table 1:** Comparison of kinetics for AO7 removal in NaClO/Ru(III)-CO_3_^2−^/HCO_3_^−^ system under different pH values with various concentrations of CO_3_^2−^/HCO_3_^−^.

Parameters	Removal Efficiency (%)		*k_obs_* (min^−1^)	R^2^	*K* (min^−1^mM^2^)	Initial–Final pH
Initial NaHCO_3_ Concentration (mM)	Within 5 min	Within 10 min				
0	33.19	46.74	0.06122	0.9815	61.2	6.89–6.88
1	68.62	86.54	0.1971	0.9952	197.1	6.85–6.88
10	73.37	88.87	0.2122	0.9852	212.2	6.85–6.80
100	81.65	91.65	0.2454	0.9787	245.4	6.90–6.89
0	71.12	92.00	0.2577	0.9983	257.7	8.50–8.49
1	77.16	96.00	0.3174	0.9967	317.4	8.50–8.48
10	89.29	100	0.4585	0.9984	458.5	8.50–8.49
100	95.03	100	0.5168	0.9909	516.8	8.49–8.49
0	61.58	78.48	0.1517	0.9898	151.7	9.98–9.66
1	62.90	80.60	0.1608	0.9909	160.8	10.01–9.98
10	63.29	81.28	0.1613	0.9893	161.3	10.02–10.02
100	71.12	86.83	0.1968	0.9884	196.8	10.01–10.01

## Data Availability

The data that support the findings of this study are available within the article and the Supplementary Information. Further data are available from the corresponding author upon reasonable request.

## Supplementary Materials

The following supporting information can be downloaded at: https://www.mdpi.com/article/10.3390/ijms26020677/s1. References [42,43,44,45,46,47,48,49,50,51,52,53] are cited in the Supplementary Materials.
