# Supplementary material for: Highly Efficient Removal of Organic Pollutants with HCO3−-Enhanced Ru(III)/NaClO Process
Source: Int J Mol Sci. 2025 Jan 15;26(2):677. doi: 10.3390/ijms26020677 (PMC11765646; doi:10.3390/ijms26020677)
Supplement: Supplementary file 1 [file ijms-26-00677-s001.zip › ijms-3418986-supplementary.pdf]

Supporting Information for

Highly efficient removal of organic pollutants with HCO_3^--enhanced Ru(III)/NaClO process

Yuhan Zhang[1,2], Guilong Peng[1,5]*, Yuting Yan[1], Xukun Meng[1], Jiangwei Zhu[3], Wenwen Gong[4,*]

[1]State Key Laboratory of Resource Insects, College of Sericulture, Textile and Biomass Sciences, Southwest University, Chongqing 400715, China

[2]Westa College, Southwest University, Chongqing 400715, China

[3]Co-Innovation Center for Sustainable Forestry in Southern China, Nanjing Forestry University, Nanjing 210037, China

[4]Institute of Quality Standard and Testing Technology, Beijing Academy of Agriculture and Forestry Science, Beijing 100097, China

[5]Yibin Academy of Southwest University, Yibin, China

*Corresponding authors.
E-mail addresses: pengguilong@swu.edu.cn (G. Peng), gong_ww@126.com (W. Gong)

Determination of the concentration of hypochlorous acid (HOCl). A colorimetric method was used to determine the concentration of HOCl according to previous method [1]. In this method, *N*,*N*-diethyl-p-phenylenediamine sulfate (DPD) was dissolved in 0.1 M H_2SO_4 to prepare a 5 g/L DPD solution. 1 mL of the sample solution was added in the mixture of 1 mL 10 mM phosphate buffer (pH = 6.5) and 1 mL of prepared DPD solution, and recorded the absorbance at λ = 510 nm using a HACH DR600 Spectrophotometer (HACH, Loveland, Colorado, USA)

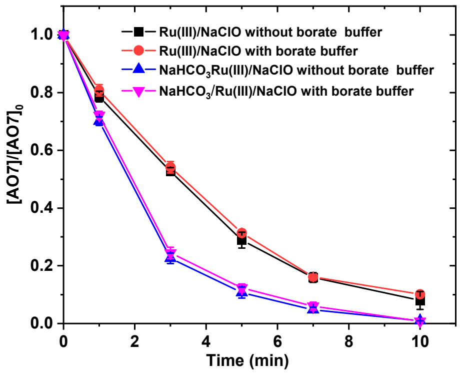

Fig. S1. The influence of borate buffer on AO7 removal in Ru(III)/NaClO process andNaHCO3/Ru(III)/NaClO process. Experimental conditions: [AO7] = 0.03 mM, [NaClO] = 0.1 mM, [Ru(III)] = 10.0 μM, pH = 8.5 ± 0.1, [$NaHCO_3$] = 10 mM; 10 mM borate buffer.

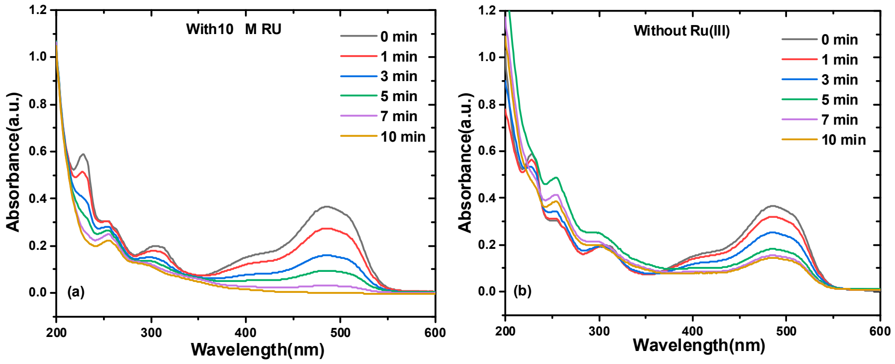

Fig. S2. UV–vis spectra of AO7 solution treated with (a) and without (b) Ru(III) activation of NaClO. Experimental conditions: [AO7] = 0.03 mM, [NaClO] = 0.1 mM, [Ru(III)] = 10.0 μM, pH = 8.5 ± 0.1, [$NaHCO_3$] = 10 mM; 10 mM borate buffer.

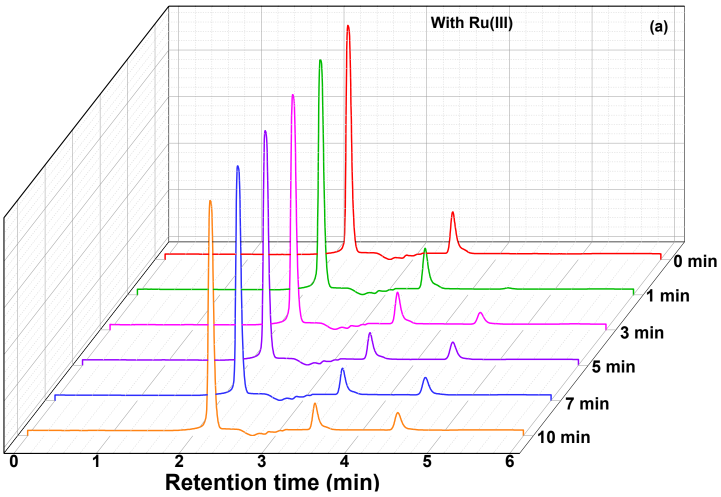

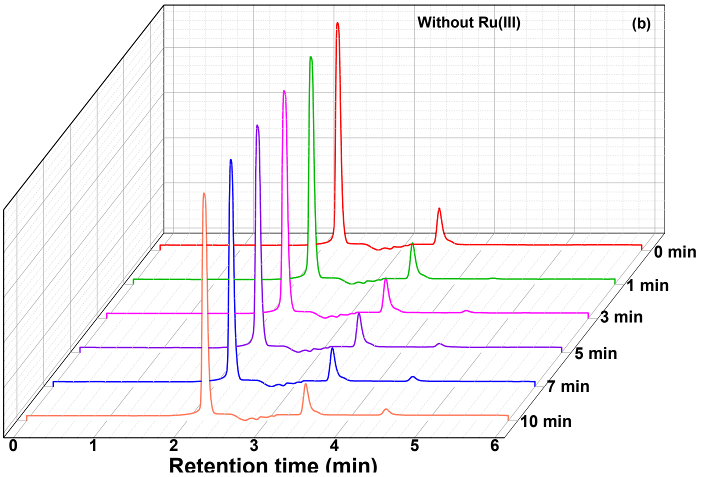

Fig. S3. HPLC chromatograms for the degradation of PMSO with Ru(III) (a) and without Ru(III) activation of NaClO. Experimental conditions: [PMSO] = 0.2 mM, [NaClO] = 0.1 mM, [Ru(III)] = 10.0 μM, pH = 8.5 ± 0.1, [$NaHCO_3$] = 10 mM; 10 mM borate buffer.

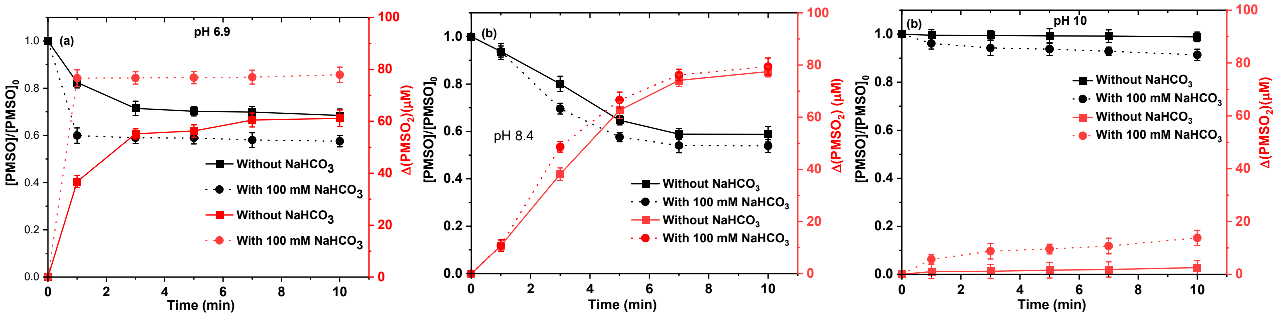

Fig. S4. Oxidation of PMSO and production of $PMSO_2$ at pH 6.9 (a), 8.5 (b) and 10.0 (c). Experimental conditions: [PMSO] = 0.2 mM, [NaClO] = 0.1 mM, [Ru(III)] = 10.0 μM, pH = 8.5 ± 0.1, [$NaHCO_3$] = 100 mM; 10 mM borate buffer.

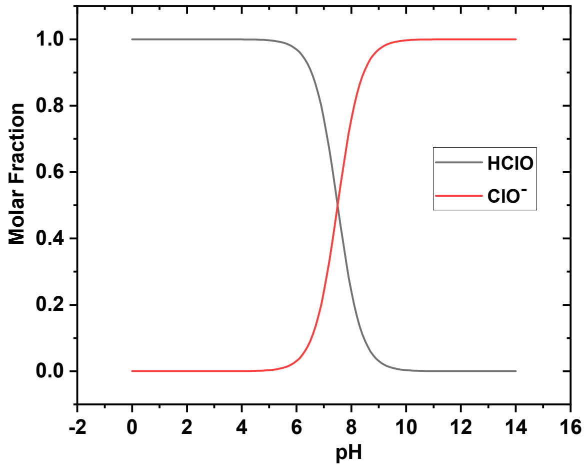

Fig. S5. Distribution of different species of HClO.

Table S1. Operating parameters for single-solute analysis of sulfamethoxazole and probe compounds by HPLC.

Compound	Mobile phase			Wavelength (nm)
	**Water with 0.2% acetic acid (%)**	**Methanol (%)**	**Flow rate (mL/min)**	
BPA	20	80	1.0	227
SMX	40	60	1.0	270
BA	40	60	1.0	228
ACT	40	60	1.0	245
p-NBA	20	80	1.0	270
Phenol	40	60	1.0	273
**Compound**	**Mobile phase**			**Wavelength (nm)**
	**Water with 0.2% acetic acid (%)**	**Acetonitrile (%)**	**Flow rate (mL/min)**	
PMSO	50	50	1.0	235
$PMSO_2$	50	50	1.0	215

Table S2. The second-order rate constants for the reaction between probe compounds, quenching agents, and micropollutants with different reactive species.

Compound	$^{•}OH$ ($M^{-1}s^{-1}$)	ClO• ($M^{-1}s^{-1}$)	Cl• ($M^{-1}s^{-1}$)	$CO_3^{•-}$ ($M^{-1}s^{-1}$)
TBA	6.0×10^8 [2]	1.3×10^7 [3]	3.0×10^8 [3]	9.6×10^4 [4]
HCO_3^-	8.5×10^6 [4]	600 [4]	2.2×10^8 [4]	
Phenol	6.6×10^9-1.8×10^{10} (pH 6-7.5) [5]	2.0×10^7 [6]	1.4×10^9 [6]	1.2×10^8[7]

ACT	1.7×10^9 [8]	-^{a}	-^{a}	1.9×10^9 [9]
DMA	-^{a}	-^{a}	-^{a}	1.8×10^9 [10]

Table S3. Summary of degradation efficiency of CBZ in various metal ions activated peroxide processes.

Metal ions	Oxidants	[Pollutants]$_0$	Removal efficiency	k_{obs} (min^{-1})	K (min^{-1}mM^2)	Ref.
Fe^{2+} (0.25 mM)	H_2O_2	0.05 mM	83%/60 min	0.19	3.04	[11]
Fe^{2+} (0.1 mM)	PDS	0.5 mM	100%/60 min	0.18	9	[12]
Fe^{3+} (0.05 mM)	PAA	0.5 mM	50%/10 min	0.1	4	[13]
Co^{2+} (0.25 mM)	PMS	0.25 mM	100%/20 min	0.25	4	[11]

a: not kinetically relevant or unknown.

Table S4. Chemical properties and structures of compounds investigated in this study.

Structure	Formula	Mol. Weight	Structure	Conformer
Sulfamethoxazole (SMX)	$C_{10}H_{11}N_3O_3S$	253.3	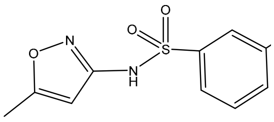	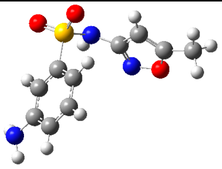
Benzoic acid (BA)	C7H6O2	122.0	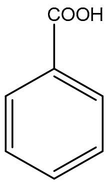	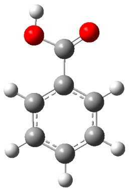
Phenol	C6H6O	94	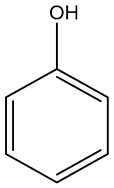	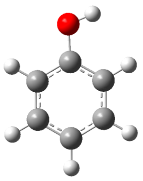
p-nitrobenzoic acid (*p*-NBA)	$C_7H_5NO_4$	167.12	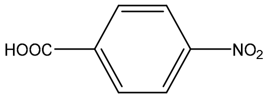	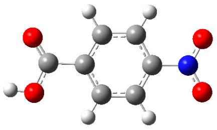
Bisphenol a (BPA)	$C_{15}H_{16}O_2$	228.29	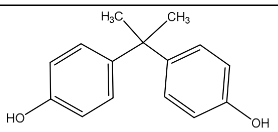	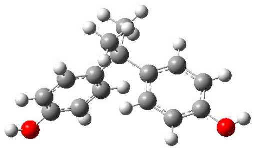

Acetaminophen (ACT)	C8H9NO2	151.17	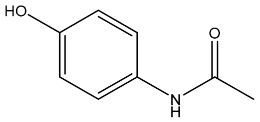	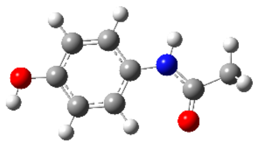

Table S5. Physico-chemical parameters of real water samples.

	pH	UV254	Cl^- (mg/L)	SO_4^{2-} (mg/L)	PO_4^{3-} (mg/L)	HCO_3^- (mg/L)	TOC (mg/L)
Ultrapure water	6.60	0	n.d.	n,d.	n.d.	n.d.	n.d.
River water	8.06	0.031	9.31	31.6	0.76	12.16	3.31
Lake wter	7.64	0.96	16.2	52.3	125.61	38.25	233.21
Tap water	8.20	0.014	24.4	22.31	0.012	33.31	1.68

References

[1] H. Xu, X. Liu, H. Li, L. Zhang, O2 activation and 1O2 generation over phosphate modified BiOCl for efficient photodegradation of organic pollutants, Applied Catalysis B: Environmental, 314 (2022) 121520.

[2] L. Gao, Y. Guo, J. Zhan, G. Yu, Y. Wang, Assessment of the validity of the quenching method for evaluating the role of reactive species in pollutant abatement during the persulfate-based process, Water Research, 221 (2022) 118730.

[3] A. Yaghoot-Nezhad, S. Wacławek, S. Madihi-Bidgoli, A. Hassani, K.-Y.A. Lin, F. Ghanbari, Heterogeneous photocatalytic activation of electrogenerated chlorine for the production of reactive oxygen and chlorine species: A new approach for Bisphenol A degradation in saline wastewater, Journal of Hazardous Materials, 445 (2023) 130626.

[4] Z. Wu, K. Guo, J. Fang, X. Yang, H. Xiao, S. Hou, X. Kong, C. Shang, X. Yang, F. Meng, L. Chen, Factors affecting the roles of reactive species in the degradation of micropollutants by the UV/chlorine process, Water Research, 126 (2017) 351-360.

[5] G.V. Buxton, C.L. Greenstock, W.P. Helman, A.B. Ross, Critical review of rate constants for reactions of hydrated electrons, hydrogen atoms and hydroxyl radicals (·OH/·O−) in aqueous solution, Phys. Chem. Ref. Data, 17 (1988) 513-886.

[6] N. Zheng, X. He, Q. Zhou, R. Wang, X. Zhang, R. Hu, Z. Hu, Generation of reactive chlorine species via molecular oxygen activation on a copper chloride loaded hydrothermal carbonaceous carbon for advanced oxidation process, Applied Catalysis B: Environmental, 319 (2022) 121918.

[7] J. Gao, R.F. Nunes, K. O'Shea, G.L. Saylor, L. Bu, Y.-G. Kang, X. Duan, D.D. Dionysiou, S. Luo, UV/Sodium percarbonate for bisphenol A treatment in water: Impact of water quality parameters on the formation of reactive radicals, Water Research, 219 (2022) 118457.

[8] Q.-Y. Wu, Z.-W. Yang, Z.-W. Wang, W.-L. Wang, Oxygen doping of cobalt-single-atom coordination enhances peroxymonosulfate activation and high-valent cobalt–oxo species formation, Proceedings of the National Academy of Sciences, 120 (2023) e2219923120.

[9] L. Wojnárovits, T. Tóth, E. Takács, Rate constants of carbonate radical anion reactions with molecules of environmental interest in aqueous solution: A review, Science of The Total Environment, 717 (2020) 137219.
[10] S.-N. Chen, M.Z. Hoffman, G.H. Parsons, Jr., Reactivity of the carbonate radical toward aromatic compounds in aqueous solution, The Journal of Physical Chemistry, 79 (1975) 1911-1912.
[11] R. Matta, S. Tlili, S. Chiron, S. Barbati, Removal of carbamazepine from urban wastewater by sulfate radical oxidation, Environmental Chemistry Letters, 9 (2011) 347-353.
[12] M.M. Ahmed, S. Chiron, Solar photo-Fenton like using persulphate for carbamazepine removal from domestic wastewater, Water Research, 48 (2014) 229-236.
[13] J. Kim, J. Wang, D.C. Ashley, V.K. Sharma, C.-H. Huang, Enhanced Degradation of Micropollutants in a Peracetic Acid–Fe(III) System with Picolinic Acid, Environmental Science & Technology, 56 (2022) 4437-4446.
